# The PPAR pan-agonist tetradecylthioacetic acid promotes redistribution of plasma cholesterol towards large HDL

**DOI:** 10.1371/journal.pone.0229322

**Published:** 2020-03-16

**Authors:** Thomas Lundåsen, Matteo Pedrelli, Bodil Bjørndal, Björn Rozell, Raoul V. Kuiper, Lena Burri, Chiara Pavanello, Marta Turri, Jon Skorve, Rolf K. Berge, Stefan E. H. Alexson, Veronika Tillander

**Affiliations:** 1 Department of Laboratory Medicine, Karolinska Institutet, Huddinge, Sweden; 2 Translational Science and Experimental Medicine, Research and Early Development, Cardiovascular Renal and Metabolism (CVRM), BioPharmaceuticals R&D, AstraZeneca, Gothenburg, Sweden; 3 Department of Clinical Science, University of Bergen, Bergen, Norway; 4 Department of Sports, Physical activity and Food, Faculty of Education, Arts and Sports, Western Norway University of Applied Sciences, Bergen, Norway; 5 Dipartimento di Scienze Farmacologiche e Biomolecolari, Centro Enrica Grossi Paoletti, Università degli Studi di Milano, Milan, Italy; 6 Department of Heart Disease, Haukeland University Hospital, Bergen, Norway; Universite du Quebec a Montreal, CANADA

## Abstract

Tetradecylthioacetic acid (TTA) is a synthetic fatty acid with a sulfur substitution in the β-position. This modification renders TTA unable to undergo complete β-oxidation and increases its biological activity, including activation of peroxisome proliferator activated receptors (PPARs) with preference for PPARα. This study investigated the effects of TTA on lipid and lipoprotein metabolism in the intestine and liver of mice fed a high fat diet (HFD). Mice receiving HFD supplemented with 0.75% (w/w) TTA had significantly lower body weights compared to mice fed the diet without TTA. Plasma triacylglycerol (TAG) was reduced 3-fold with TTA treatment, concurrent with increase in liver TAG. Total cholesterol was unchanged in plasma and liver. However, TTA promoted a shift in the plasma lipoprotein fractions with an increase in larger HDL particles. Histological analysis of the small intestine revealed a reduced size of lipid droplets in enterocytes of TTA treated mice, accompanied by increased mRNA expression of fatty acid transporter genes. Expression of the cholesterol efflux pump *Abca1* was induced in the small intestine, but not in the liver. *Scd1* displayed markedly increased mRNA and protein expression in the intestine of the TTA group. It is concluded that TTA treatment of HFD fed mice leads to increased expression of genes involved in uptake and transport of fatty acids and HDL cholesterol in the small intestine with concomitant changes in the plasma profile of smaller lipoproteins.

## Introduction

The synthetic saturated fatty acid tetradecylthioacetic acid (TTA) is comprised of a 16-carbon backbone with an insertion of a sulfur atom in position 3 (β-position) from its carboxyl end. This chemical modification still allows TTA to be absorbed in the intestine and transported to the liver where it can act as substrate for desaturation and incorporation into glycerolipids, preferably into the phospholipid fraction [1]. Although TTA has physicochemical properties similar to natural fatty acids, the sulfur-substitution blocks β-oxidation of the fatty acid from the carboxyl end. The relatively slow hepatic and renal occurring metabolism of TTA instead involves ω-oxidation followed by partial β-oxidation from the omega end [2–4]. TTA has been shown to be a potent ligand for nuclear receptors of the peroxisome proliferator-activated receptor (PPAR)-family [3–9]. Resembling other PPARα agonists, TTA administration to rodents has a pronounced plasma triacylglycerol (TAG) reducing effect [10,11]. In addition some hypolipidemic effects of TTA have been demonstrated to be partly PPARα independent, thus supporting the hypothesis that TTA acts as a PPAR pan-ligand activating also PPARγ and δ [5,9]. In addition to the mentioned plasma TAG reducing effect, TTA promotes a multitude of biological effects that are mostly considered beneficial for health. For example, TTA has been shown to reduce body weight gain (especially by reducing adiposity), to provide beneficial effects on insulin resistance and elevated plasma glucose levels, and to improve dyslipidemia in pre-clinical models of obesity and diabetes [8,12–14]. TTA has also been proven to have anti-oxidative and anti-inflammatory effects both *in vivo* [14–17] and *in vitro* [15,17] Despite all these evidence, no study has investigated in details the effect of TTA on plasma lipoprotein and the role of the intestine in their modulation. The aim of the present study was to investigate TTA dependent effects on lipid and lipoprotein metabolism in the small intestine and liver in mice fed HFD. Analysis of plasma lipids revealed that TTA treatment lowers plasma TAG and led to a redistribution of total plasma cholesterol into large HDL particles. In line with these findings, TTA increased expression of the HDL cholesterol transporter *Abca1* mRNA in the small intestine. TTA treatment also induced several genes with functions in fatty acid activation and transport and decreased the expression of genes involved in lipogenesis. Histological analysis of intestine showed a dramatic decrease in lipid droplet size in the enterocytes together with a robust increase of the fatty acid desaturating enzyme SCD1 in the villi of mice receiving TTA.

## Materials and methods

### Animals and diets

Ten-week-old C57BL/6 male mice (purchased from Taconic, Ry, Denmark, and allowed one week of acclimatization on chow diet before study start) were fed either a high fat control diet (HFD, n = 9), or a high fat diet containing 0.75% TTA (n = 9), and water *ad libitum* for 6 weeks. The HF diet contained 24% fat w/w (21,3% lard and 2,3% soy oil). Mice were housed in groups of 3 per cage in open cages at a constant temperature of 22°C and a dark/light cycle of 12h/12h. Cumulative food intake was recorded three times during the study by weighing the remaining food one or two days after food supply. Body weight was recorded for each mouse every seventh day. Mice were anesthetized using 2% Isoflurane (Schering-Plough, Kent, UK), followed by cardiac puncture, the blood was collected and EDTA-plasma was prepared by centrifugation and frozen at -20°C until further analysis. Livers were collected and snap frozen in liquid nitrogen and stored at -80°C. For histology, the small intestine was excised (n = 3 per group) and fixed in 4% formaldehyde overnight and then stored in 70% ethanol until further processing. Intestines from the rest of the animals were processed at +4 ^o^C, excised, rinsed with cold phosphate buffered saline, divided into four segments of equal length and cut open. The intestinal mucosa cell layer was scraped off and transferred to TRI Reagent® (Applied Biosystems, Carlsbad, CA, USA) and the tubes were directly frozen in liquid nitrogen and stored at -80°C until further analysis.

The animal experiments were carried out with ethical permission obtained from the Norwegian State Board for Biological Experiments (Project number 20091741) and followed the Norwegian Research Councils ethical guidelines, the Guide for the Care and Use of Laboratory Animals, and the Guidelines of the Animal Welfare Act.

### Liver lipids

Total liver lipids were extracted using the method of Bligh and Dyer [18]. Solvents were evaporated and samples were then dissolved in isopropanol before enzymatically measured on a Hitachi 917 system (Roche Diagnostics, Mannheim, Germany) using the triacylglycerol (TAG)(GPO-PAP) and cholesterol kits (CHOD-PAP) from Roche Diagnostics, and the phospholipid (PL) kit from bioMérieux SA (Marcy l'Etoile, France).

### Plasma lipid and lipoprotein analyses

Plasma lipoproteins were separated from 2,5 μl plasma by size exclusion chromatography (SEC) and lipids (TAG and cholesterol) were quantified with a real-time detection method. Plasma total cholesterol and TAG concentration were calculated by integration of the areas under the curves from each individual profile [19,20].

To further study the plasma lipoproteins, equal amounts of plasma from each animal from respective groups were pooled and lipoproteins were then separated using SEC or separated using D_2_O/sucrose density gradient ultracentrifugation as previously described [21]. Fifteen μl of every second minute (from minute 39 to 55) fraction after the SEC separation or 5 μl of lipoprotein isolated by density gradient centrifugation were separated using a 7–15% gradient SDS-polyacrylamide gel (BioRad Laboratories, Hercules, CA, USA) under denaturing conditions. Proteins were transferred onto nitrocellulose filters (Nitropure, Micron Separations Inc., Westborough, MA, USA), and blots were probed with antibodies against ApoA1 (Goat anti-Human ApoA1, Rockland antibodies and assays, Gilbertsville, PA) and ApoE and ApoB (Rabbit anti-Mouse ApoE and ApoB, Meridian LifeScience,^®^ Inc. Memphis, TN, USA), followed by detection by IRDye^TM^ 800 anti-Goat IgG and IRDye^TM^ 680 anti-Rabbit IgG antibodies using The Odyssey^®^ Imaging System (LICOR, Lincoln, NB, USA). Native lipoprotein gel electrophoresis analysis was performed using a semi-automated agarose gel electrophoresis system (Hydrasis, SEBIA Inc, 400–1705 Corporate Drive, Norcross, GA 300, USA), using Hydragel 7 LIPO+Lp(a) and lipoproteins were stained by Sudan black dye.

Serum HDL subclasses were separated by 2D electrophoresis, in which agarose gel electrophoresis was followed by non-denaturing polyacrylamide gradient gel electrophoresis and subsequent immunoblotting with anti-mouse apoA-I (Rockland, Gilbertsville, PA, USA) or an anti-mouse apoE (Calbiochem, Merck, Darmstadt, Germany)[20,22]. Plasma ApoA1 levels were detected by ELISA using the Mouse Apolipoprotein A1 ELISA ^PRO^ kit (MABTECH AB, Nacka Strand, Sweden) following the manufacturer´s protocol.

### RNA isolation and cDNA synthesis

Livers and intestinal epithelium were used for gene expression analysis. Tissues were homogenized and total RNA was isolated using the MagMax total RNA isolation system (Applied Biosystems, Carlsbad, CA, USA). RNA quantity was measured using spectrophotometry (NanoDrop 1000, NanoDrop Technologies, U.S.A), and quality control for each sample was performed using the Experion Automated Electrophoresis System (BioRad). The quality limit for RNA before further analysis was set to R/Q value of 7 (out of 10). cDNA was synthesized using pooled or individual RNA samples (500 ng RNA per reaction) using High Capacity RNA to cDNA Mastermix (Applied Biosystems).

### Gene expression analysis

Two types of TaqMan Low Density Arrays (TLDA) in 96-well formats (format 96b, Applied Biosystems) were designed to investigate the expression of genes related to peroxisomal and mitochondrial metabolic pathways. The TLDAs were run at the Bioinformatics and Expression Analysis core facility (BEA) at Karolinska Institutet and mRNA expression data were analyzed by RQI Manager (Applied Biosystems). Gene expression was calculated using the 2^-ΔΔCt method using *18S* as a reference gene. For QPCR on individual samples a mean for two reference genes Hypoxyxanthine phosphoribosyltransferase 1 (*Hprt*) and *18S*, and *Cyclophilin* and 18S were calculated and used for intestinal and liver samples respectively.

### Lipolytic activity in tissues

White adipose tissue (WAT) and skeletal muscle were homogenized in tissue homogenization buffer (150mM NaCl, 10mM Tris-HCl, 2mM EDTA, pH 7,4) supplemented with complete protease inhibitor (Sigma-Aldrich, Merk KGaA, Darmstadt, Germany) and phosphoSTOP (Sigma-Aldrich) using a Teflon douncer. Homogenates were left on ice for 30 min before centrifugation at 10 000xg for 10 min at 4°C following supernatant collection. Protein concentration was determined and 3 and 5 μg of total tissue of WAT and skeletal muscle, respectively, were used for lipase activity using Lipoprotein Lipase (LPL) Activity Assay Kit (Roar Biochemical Sigma-Aldrich). The described enzymatic fluorescence assay is not specific for LPL and detects lipase activity in tissue [23].

### Immunoassay

White adipose tissue was homogenized in homogenization buffer (150mM NaCl, 10mM Tris-HCl, 2mM EDTA, pH 7,4) and liver tissue in RIPA-buffer (Radioimmunoprecipitation assay buffer 150 mM NaCl, 2mM EDTA, 0,35% Na-deoxycholate, 0,50% Nonidet-40, 0,10% SDS both buffers were supplied with complete protease inhibitor (Sigma-Aldrich, Merk KGaA, Darmstadt, Germany) and phosphoSTOP (Sigma-Aldrich) using a Teflon douncer or a bead beater. Samples were incubated on ice for 30 min and further centrifuged at 10 000xg for 10 min at 4°C and the supernatants were collected.

Total protein concentration was determined using Bradford assay (BioRad). Total proteins were separated using 10 or 12% gradient SDS-polyacrylamide gel (BioRad) under denaturing conditions. Proteins were transferred onto nitrocellulose filters (Nitropure), and blots were probed with antibodies against phosphorylated and total HSL, GAPDH, ASCL1 (Cell Signaling Technology, Inc, Danvers, MA, USA) and FATP2 (abcam, Cambridge, UK), followed by detection by IRDye^TM^ 800 anti-Goat IgG and IRDye^TM^ 680 anti-Rabbit IgG antibodies using The Odyssey^®^ Imaging System (LICOR).

### Immunohistochemistry

4 μm paraffin section were mounted on charged glass slides (Superfrost® Plus, Thermo Scientific, Menzel-Gläser) and pretreated with Rodent decloaker buffer (BioCare) at pH 6 in a 2100 automated pressure cooker (PickCell). Consecutive sections were incubated overnight with a polyclonal guinea pig anti-mouse Perilipin2 (Progen, GP40) in 1:2000 dilution, and monoclonal rabbit anti-mouse SCD1 (Cell Signaling #2794) in dilution 1:100. For the Perilipin2 antibody a mouse on mouse detection kit (Vector, BMK-2202) was used according to the manufacturer’s protocol. Anti-Scd1 incubated sections were blocked in 4% normal goat serum and incubated with biotinylated goat anti rabbit (Dako, E0432) at a 1:300 dilution. Immunoreactivity was visualized using routine avidin-biotin amplification and diaminobenzidine (DAB) chromogenic reaction.

### Statistical analyses

All results are presented as mean ± SEM and Student´s t test was used for analysis of difference, significant changes between groups are indicated in figures and tables.

All statistics were calculated using GraphPad Prism 5.0d.

## Results

### TTA attenuates HFD induced body weight gain but not HFD induced fatty liver

**Fig 1:**
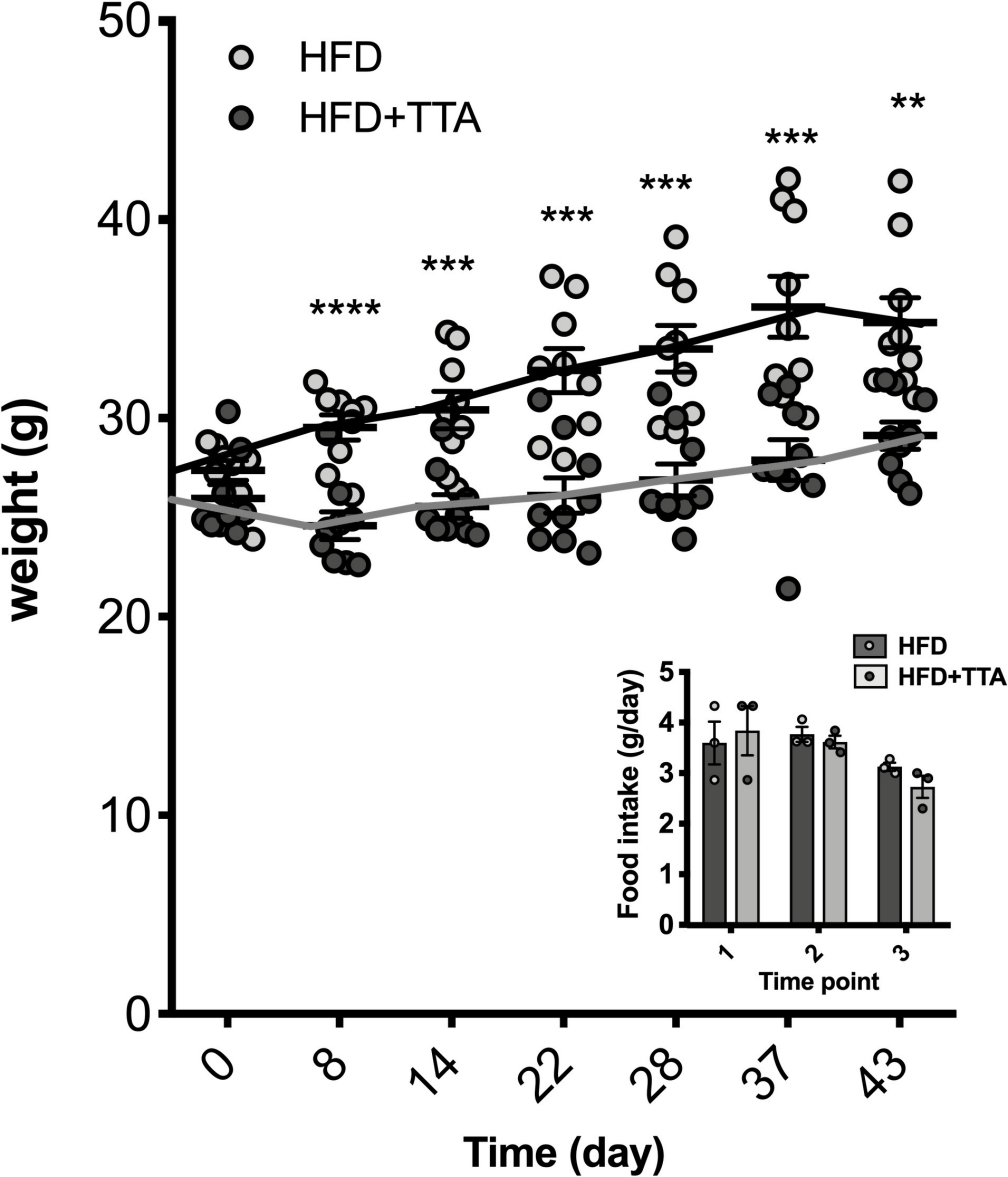
Body weight.

Age matched male C57BL/6J mice were fed HFD as control or HFD supplemented with 0.75% TTA for six weeks. In line with previous findings the TTA group displayed an attenuated body weight gain (Fig 1) without any change in food intake with time or between the groups.

Mice were weighted weekly, body weights of HFD controls (open circles, n = 9) and HFD+TTA treated mice (grey boxes, n = 9). Imbedded bar graph represents the mean food intake per mouse per day in the three cages (n = 3) at the different three time points. Data shown are mean ± SEM, and student’s unpaired t-test was performed for each data set. * = p<0.05, ** = p<0.01, *** = p<0.001 and **** = p<0.0001.

The final body weight of the TTA treated mice was approximately 4 grams lower than that of the control group. However, the reduced body weight was not accompanied by a decrease in liver weight. Instead, TTA increased both total and relative liver weight (Table 1). TTA treatment elevated relative (μmol/g) hepatic TAG and PL levels, but not cholesterol levels (Table 1).

**Table 1:** Liver weight and lipid content.

	HFD (mean ± SEM)	HFD+TTA (mean ± SEM)
**Liver weight (g)**	1.6 ± 0.1	2.1 ± 0.1 ***
**Body to liver weight (%)**	4.7 ± 0.1	7.2 ± 0.2 ****
**Liver Cholesterol (umol/g)**	5.7 ± 0.2	6.0 ± 0.2
**Liver TAG (umol/g)**	20.4 ± 4.9	37.6 ± 3.4 *
**Liver PL (umol/g)**	18.4 ± 0.5	21.9 ± 0.3 ****

Data shown are mean ± SEM, and student’s unpaired t-test was performed for each data set

* = p<0.05

*** = p<0.001 and

**** = p<0.0001.

### TTA effectively decreases plasma TAG levels and causes redistribution of plasma cholesterol

Confirming previous results, a 3-fold decrease in total plasma TAG in TTA treated mice was observed (Table 2).

**Table 2:** Plasma lipids.

Plasma lipids (mmol/L)	HFD (mean ± SEM)	HFD+TTA (mean ± SEM)
**Triacylglycerol**		
Total	1.07 ± 0.07	0.36 ± 0.09 ***
VLDL/remnants	0.68 ± 0.10	0.15 ± 0.06 **
LDL	0.29 ± 0.03	0.10 ± 0.02 ***
HDL	0.10 ± 0.03	0.11 ± 0.03
**Cholesterol**		
Total	3.42 ± 0.10	3.49 ± 0.32
VLDL/remnants	0.09 ± 0.01	0.04 ± 0.01 **
LDL	0.28 ± 0.09	0.87 ± 0.19 *
HDL	3.04 ± 0.17	2.58 ± 0.17

**Fig 2:**
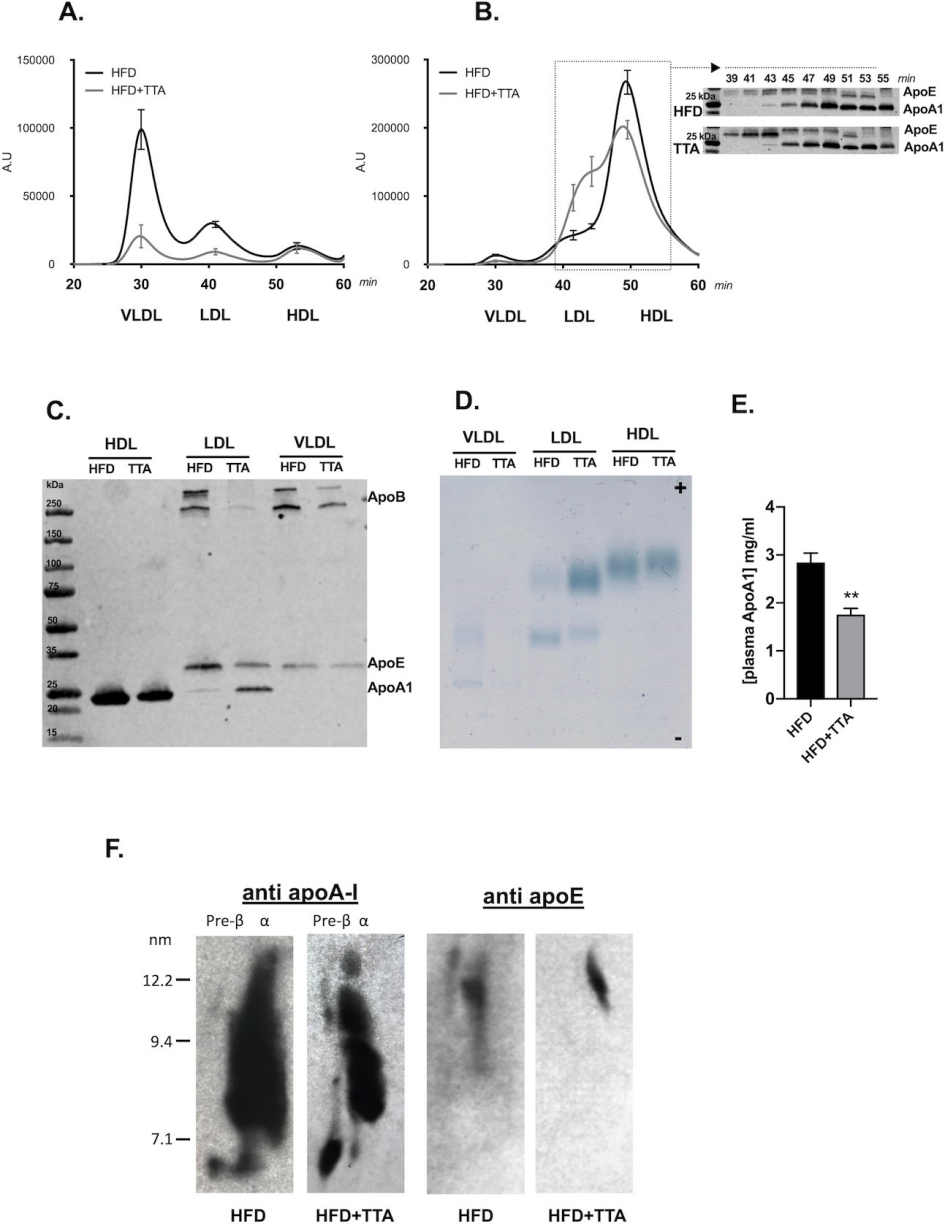
Plasma cholesterol and triacylglycerol lipoprotein profiles. **a.** Plasma triacylglycerol lipoprotein profiles. The fig shows the triacylglycerol lipoprotein profiles of plasma samples from HFD control mice (black line, n = 5) and HFD+TTA treated mice (grey line, n = 5) separated by SEC. The first peak in the graph represents VLDL/chylomicron remnants-sized particles, the second peak LDL-sized particles and the last peak HDL-sized particles. Curves represent the mean value of five individual samples from each group, and for each peak one time-point was chosen to show the SEM of the group. **b.** Plasma cholesterol lipoprotein profiles. The figure shows the cholesterol lipoprotein profiles for HFD controls (black line, n = 5) and HFD+TTA treated mice (grey line, n = 5). The first peak in the graph corresponds to VLDL/CM-sized particles, the second peak LDL-sized particles and the last peak HDL-sized particles. Curve represent the mean value of five individual samples from each group, and for each peak one time point (30 min for VLDL/remnants, 41 min for LDL and 50 min for HDL) was chosen to show the SEM of the group. An extra time point was included at 43 min, between the HDL and the LDL peak to emphasize the changed cholesterol profile in the HF+TTA group. Immunoblots below the graph show representative western blots of ApoE and ApoA1 from SEC-separated pooled samples from each group. One-minute fractions were collected and representative fractions for the “beginning of LDL sized particles” to HDL sized particles were chosen, separated on a gradient gel and used for western blot. Upper blot shows fractionated plasma from pooled HF plasma. Lower blot shows fractionated plasma from pooled HF+TTA plasma. Marker to the left followed by every second fractions from minute 39 to 55, this section is also marked with a line in the cholesterol lipoprotein profiles. **c.** Western blot analysis of lipoproteins in fractionated plasma. Equal amounts of plasma from each animal in respective group were pooled and separated using density gradient ultracentrifugation and 5 μl of each fraction (VLDL/remnants, LDL and HDL) were separated on a gradient gel and used for Western blot analysis of ApoB (100 and 48), ApoE and ApoAI. **d.** Native gel electrophoresis analysis of plasma lipoproteins. VLDL/remnants, LDL and HDL fractions isolated by ultracentrifugation from pooled plasma samples were separated by agarose gel electrophoresis and lipoprotein bands were stained by Sudan Black dye. **e.** Total plasma ApoA1 content determined by ELISA (n = 5). **f.** Western blot analysis of lipoproteins in HDL fraction separated by 2-D gel electrophoresis.

The plasma lipids were calculated from the SEC analysis shown in Fig 2A and 2B. Data shown are mean ± SEM, and student’s unpaired t-test was performed for each data set

* = p<0.05

** = p<0.01 and

*** = p<0.001.

Analysis of the TAG content in the different lipoprotein particles after separation by SEC revealed the TAG reduction by TTA treatment was due to a 4.5-fold and a 3-fold reduction of TAG in the VLDL/remnant and LDL fractions, respectively (Fig 2A).

Total plasma cholesterol levels did not change (Table 2), but the TTA treatment led to a clear redistribution of cholesterol among the different lipoprotein particles (Fig 2B). Cholesterol in the VLDL/remnant fraction was reduced about 2-fold and cholesterol levels in the HDL peak was not changed. In contrast, LDL cholesterol seemed to be increased by the treatment. Interestingly, analysis of the cholesterol lipoprotein profile revealed the appearance of a new lipoprotein fraction in the plasma of TTA mice. These particles had a peak appearing between 43 and 45 min, a retention time in between that of the LDL and HDL peaks. To characterize the nature of this generated lipoprotein particle subgroup, we collected the lipoprotein fractions appearing between 39–55 minutes, which were subjected to SDS-PAGE followed by immunoblotting against ApoE and ApoA1 (see Fig 2B). The lipoprotein fractions from TTA treated mice eluted at 41 and 43 and showed higher content of ApoE compared to those from non-treated animals. The content of ApoAI started to increase in TTA treated animals in in the fraction collected at 45 min. This result suggested the appearance of large HDL particles in response to TTA treatment. To further characterize these particles, lipoproteins were also separated by D_2_O-sucrose sequential density gradient ultracentrifugation from pooled plasma of HF or HF+TTA fed mice and the ApoAI, ApoB and ApoE contents were quantified by Western blot analysis (Fig 2C). The ApoE content was similar in the VLDL/remnants fractions from control and TTA treated mice, while ApoB was reduced by TTA treatment in this lipoprotein fraction. The presence of ApoE was also evident in the LDL fraction with stronger intensity in the control group compared to the TTA treated group. An increased amount of ApoAI was observed in the LDL fraction isolated from TTA plasma compared to HFD controls, indicating the presence of large HDL particles floating at the same density range. Moreover, ApoB (48 and 100) was strongly decreased in the VLDL/remnant and LDL fractions by the TTA treatment. The ApoAI content in the HDL fraction appeared to be decreased in the TTA group, but increased in the LDL fraction. We performed a native agarose gel electrophoresis analysis on the isolated lipoproteins, followed by Sudan Black staining (Fig 2D). The VLDL particles migrating in pre-beta position were reduced in the TTA group, confirming the results of lipid quantification by SEC analysis. The native agarose gel electrophoresis also revealed the presence of two bands in the LDL fraction; one band migrating in beta position typical for LDL particles, and a second band migrating in alpha position specific for HDL particles. Importantly, the intensity of the LDL band was reduced in TTA treated animals, whereas the intensity of the HDL band was higher in these mice when compared to the control animals. Plasma ApoA1 was reduced in TTA treated animals compared to control (Fig 2E). The TTA treated mice showed an enriched proportion of small pre-β HDL. and seemed to carry more large ApoE-containing HDL with a diameter above 12 nm (Fig 2F).

### TTA induces lipolysis in adipose tissue and fatty acid uptake and metabolism in liver

**Fig 3:**
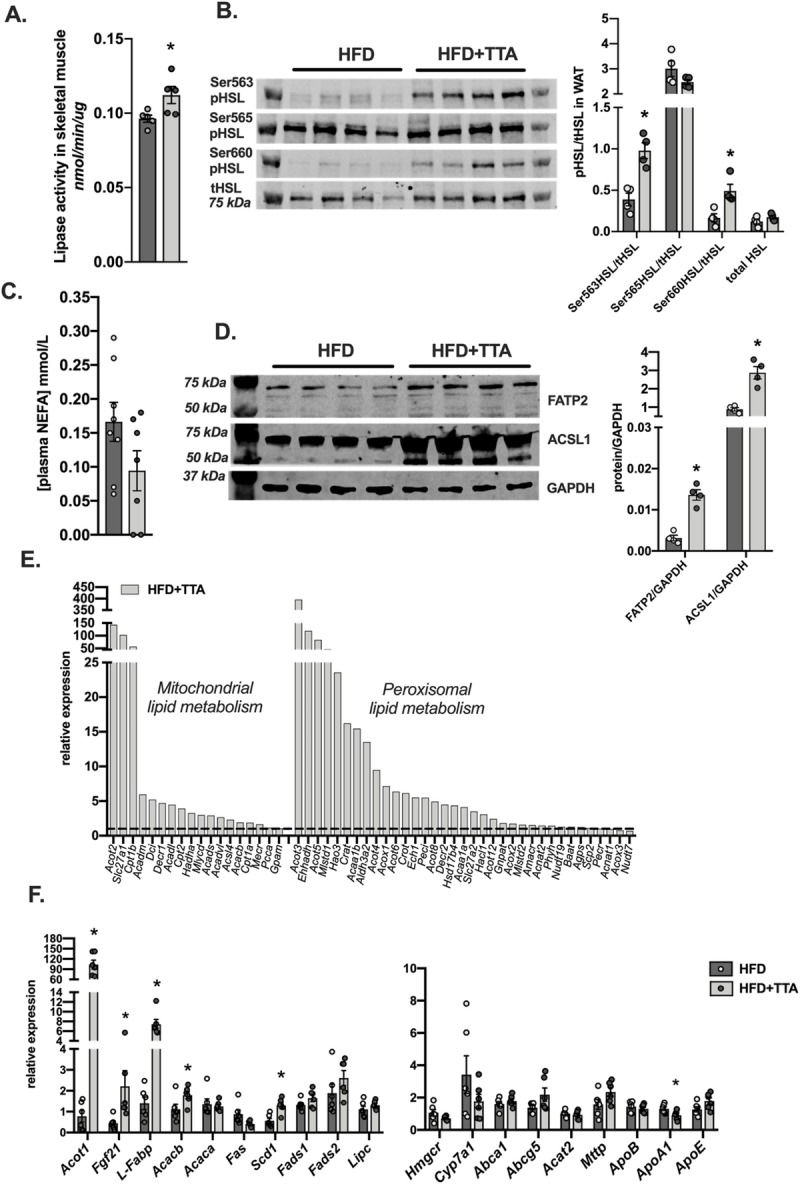
Regulation of genes in hepatic lipid metabolism. **a.** Total lipase activity in skeletal muscle lysate from control and TTA fed mice (n = 5). **b**. Immuno blot analysis and quantification of phosphorylated HSL and total HSL in white adipose tissue from control and TTA fed mice (n = 4). **c.** Total plasma NEFA (non-esterified fatty acids) (n = 8 and 7 for HFD and HFD+TTA respectively). **d.** Gene regulation of hepatic mitochondrial- and peroxisomal-lipid metabolism genes. mRNA expression data from TaqMan Low Density Arrays of pooled samples from each group (n = 6). Dashed line represents the expression of the HFD-pooled sample normalized to 1. *18S* was used as reference gene. **e.** Selected genes involved in fatty acid synthesis and cholesterol metabolism were analyzed in individual samples (n = 6) from HFD and from HFD+TTA, with one individual sample in the HFD control group used as calibrator. Data in bar plots are shown as mean ± SEM, in HFD as dark grey bars with individual values in light grey circles and in HFD+TTA as light grey bars with individual values in dark grey circles. Student´s unpaired t-test was performed on each data set, * = p<0.05 and *** = p<0.001.

Since TTA decreased body weight and that a reduced adiposity was observed at animal sacrifice, the rate of total lipase activity was investigated in skeletal muscle and white adipose tissue (WAT). While total lipase activity was increased in skeletal muscle by TTA supplementation (Fig 3A), no change was seen in adipose tissue when comparing the two groups. However, when investigating the phosphorylation status of HSL (as an indicator of increased lipolysis) in WAT, two out of three sites on HSL were highly phosphorylated in TTA treated mice compared to control after overnight fast (Fig 3B). Increased lipolysis would result in increased amount of NEFA in the plasma, however no increase in NEFA were detected in TTA treated mice (Fig 3C). An increased uptake of fatty acid by the liver would theoretically drain the plasma of NEFA and also contribute to the increase seen in liver TAG in mice treated with TTA. In support of this, the protein expression of Fatty acid transporter 2 (FATP2), a protein important for fatty acid uptake in hepatocytes, and the Acyl-CoA syntethase long 1 (ACSL1), a mitochondrial associated acyl-CoA synthase known to be important both for proper fatty acid oxidation and incorporation into glycerophospholipids, were increased in liver by TTA (Fig 3D). To gain further insight into the effects of TTA on intracellular lipid metabolism in the liver, genes important for mitochondrial and peroxisomal lipid metabolism were investigated. Several genes were dramatically induced in the TTA treated animals compared to controls (Fig 3E).

In particular, the mitochondrial PPARα target genes *Acot2* (Acyl-CoA thioesterase 2), *Slc27a1* (*Fatp1*, fatty acid transporter protein 1) and *Cpt1b* (Carnitine palmitoyl transferase 1b), were increased 60-140-fold in TTA-treated livers (Fig 3E, left panel). In addition, several genes involved in mitochondrial β-oxidation of fatty acids were induced 2 to 5-fold in the livers of the TTA group, e.g. *Cpt2* (Carnitine palmitoyl transferase 2), *Acadm* and *Acadl* (Medium- and Long-chain acyl-CoA dehydrogenase), *Dci* (Mitochondrial delta 3, delta 2-enoyl-CoA isomerase), *Decr1* (2,4-dienoyl-CoA reductase) and *Hadha* (the alpha subunit of mitochondrial Trifunctional protein) (Fig 3E).

Similarly, several PPARα target genes involved in peroxisomal lipid metabolism displayed marked induction of their mRNA levels upon TTA treatment, including *Acot3* (Acyl-CoA thioesterase 3), *Ehhadh* (Enoyl-coenzyme A hydratase/3-hydroxyacyl coenzyme A) and *Acot5*(Acyl-CoA thioesterase 5) with 80-400-fold induction (Fig 3A, right panel). In addition, 17 other genes with functions in peroxisomal fatty acid metabolism were induced 2-20-fold in the TTA treated group, including all genes coding for proteins involved in peroxisomal β-oxidation of straight chain fatty acids.

The cytosolic Acyl-CoA thioesterase 1 (*Acot1*) was strongly induced by TTA treatment, as expected since it is an established PPARα target gene [24]. *Fgf21* (fibroblast growth factor 21), another reported PPARα target gene [25,26] and hormonal mediator of fatty acid oxidation and lipid metabolism, increased with the TTA treatment (Fig 3F).

No changes in *Acaca* mRNA, and a tendency (p = 0.0501) of *Fasn* mRNA to be down regulated were noted. However, mRNA for *Scd1* coding for the fatty acid modulating enzyme Stearoyl-CoA desaturase 1 was increased with the TTA treatment (Fig 3F).

In view of the drastic changes in the plasma lipoprotein fractions in the TTA treated group, a selected set of mRNAs coding for proteins involved in hepatic cholesterol metabolism and transport were quantified. However, except for a small decrease in the expression of *ApoA1*, none of these gene transcripts (*Hmgcr*, *Cyp7a1*, *Abcg5*, *Abca1*, *Acat2*, *Mttp*, *ApoB and ApoE*) were significantly changed between the groups (Fig 3F).

### TTA effects gene expression and reduces the size of lipid droplets in the enterocytes

The uptake and processing of lipids in the small intestine has a major impact on systemic lipid homeostasis. The small intestine was analyzed for TTA mediated regulation of gene expression by QPCR and for changes in lipid storage by immunohistochemistry.

**Fig 4:**
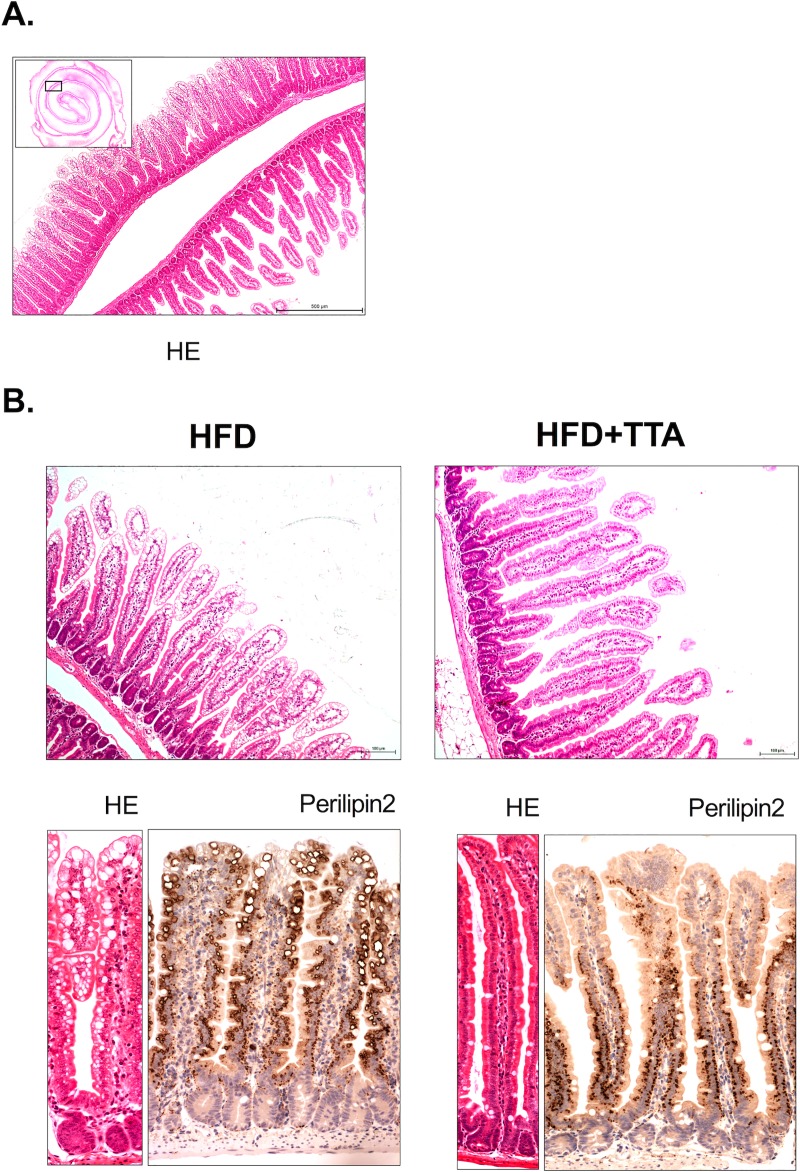
Lipid droplets in small intestines from HFD controls and HFD+TTA fed mice. **a.** Inserted picture show an overview of a cross section of the proximal half of a hematoxylin-eosin stained intestine from a HFD fed control mouse. The box represents the magnified area shown to the right, showing a proximal part of the intestine without evident lipid droplets and one adjacent part, located more to the middle section of the intestine, in which the lipid droplet accumulation in the luminal part of the villi is evident. **b.** Upper row shows representative pictures of hematoxylin-eosin stained intestines at 20x magnification, from approximately 5 cm in from the beginning of the duodenum, from HFD control and from HFD+TTA treated mice. Lipid droplets are abundant in mice fed HFD and abolished in mice fed the TTA supplemented diet.

Fig 4A shows a hematoxylin-eosin stained section of the proximal half of the intestine from a HFD fed control mouse, and in higher magnification two adjacent parts of the intestine in which the most proximal part has low amount of lipid droplets and the more adjacent part located further away from duodenum where lipid droplets are more evident.

Lower row shows representative pictures of hematoxylin-eosin stained and Perilipin2 antibody labeled intestines at 40x magnification for HFD (left) and HFD+TTA (right).

As seen from Fig 4B, the lipid accumulation was largely abolished in the mid sections of the small intestine of the TTA treated group compared to control mice. The vacuoles were confirmed to be lipid-containing vesicles by immunohistochemistry against the peripheral lipid vesicle membrane component Perilin2 (Fig 4B).

**Fig 5:**
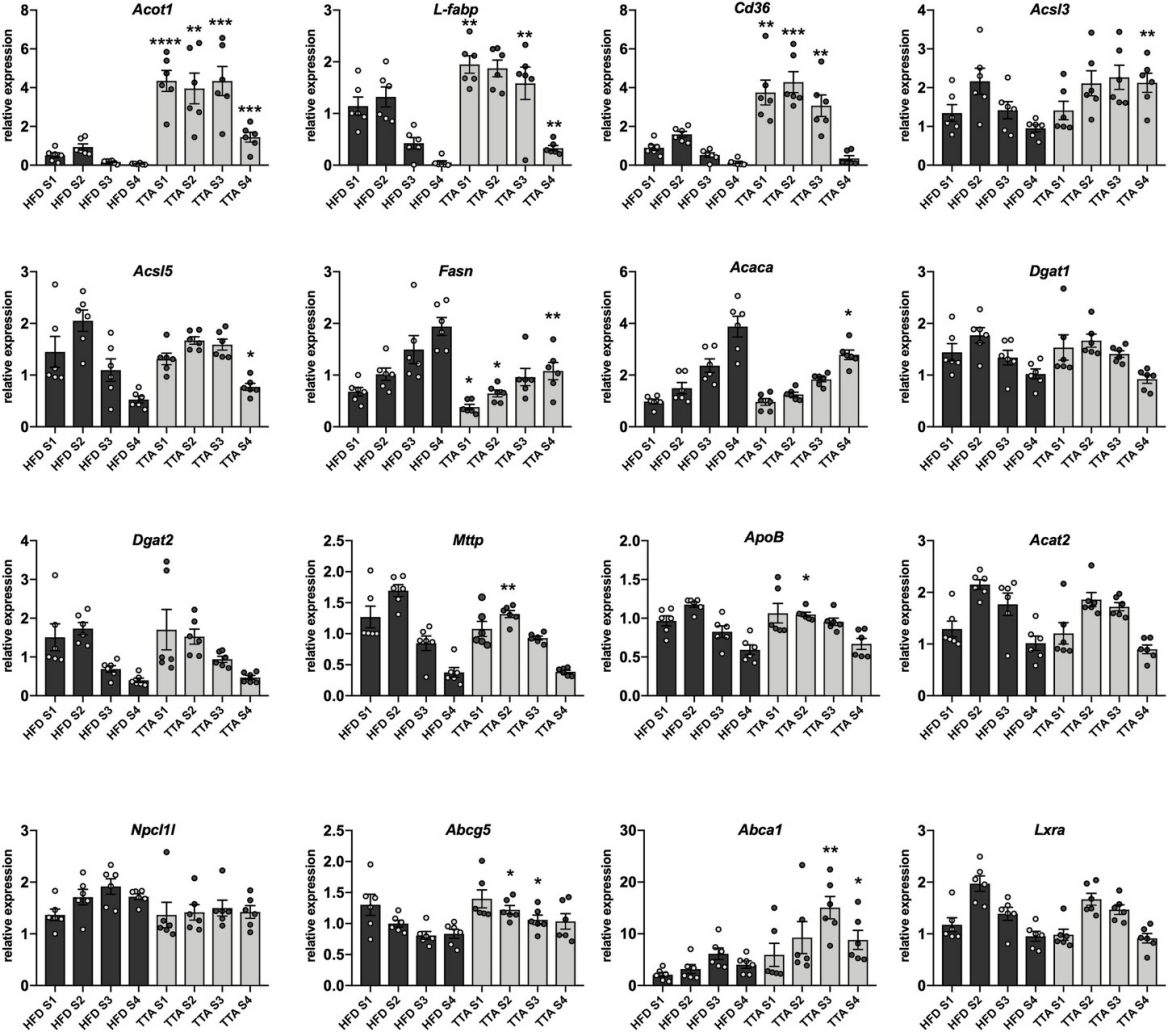
Regulation of genes involved in intestinal fatty acid and cholesterol metabolism. The intestine was divided into 4 segments of equal length, S1 (most proximal) to S4 (most distal). Genes coding for proteins involved in intestinal fatty acid and cholesterol metabolism were analyzed in individual samples (n = 6). S1 of one individual in the HFD control group was used as a calibrator to also visualize the pattern of gene expression throughout the intestine. Data in bar plots are shown as mean ± SEM, in HFD as dark grey bars with individual values in light grey circles and in HFD+TTA as light grey bars with individual values in dark grey circles and Student´s unpaired t-test was performed on each data set for each segment * = p<0.05, ** = p<0.01, *** = p<0.001 and **** = p<0.0001.

Messenger RNA expression of a selected set of genes involved in fatty acid and cholesterol metabolism in the mucosa layer of the small intestine were further investigated by QPCR. For this purpose, the small intestine was divided into four segments of equal length, denoted S1 (proximal) through S4 (distal segment). PPARα activation was evident in the intestine, as *Acot1* mRNA expression was highly increased (induced 8–20 fold) throughout the intestine, with the highest expression in the proximal and middle part of the small intestine, a pattern that is similar to the pattern of PPARα expression in the intestine [24]. PPARα target genes fatty acid translocase *Cd36* and fatty acid binding protein 1 (*L-Fabp*) expression were induced approximately 2-3-fold in the S1 and S2 segments of the TTA treated animals (Fig 5). Acyl-CoA synthetase 3 and 5 (*Acsl3* and *Acsl5*) were also increased in the TTA treated group, however, the induction was only evident in the distal part of the intestine (Fig 5).

Differences in endogenous fatty acid synthesis in the small intestine may theoretically contribute to the observed differences in lipid accumulation in the intestinal epithelium in TTA treated mice. Accordingly, the genes coding for Acetyl-CoA carboxylase (*Acaca*) and Fatty acid synthase (*Fasn)* were significant decreased in their mRNA expression by TTA (Fig 5). *Dgat1* and *Dgat2* (Diacylglycerol acyltransferases 1 and 2), *Mttp* (Microsomal triglyceride transfer protein) and *ApoB* are essential in the process of lipid droplet and CM formation in the intestine. Of these genes were only *ApoB* and *Mttp* were slightly, but significantly, decreased by TTA, in segment S2 (Fig 5). The mRNA expression levels of *Npc1l1*, *Abcg5*, *Acat2*, and *Abca1*, involved in cholesterol transport and esterification in the small intestine, were also analyzed. Of these, *Npc1l1* and *Acat2* mRNA levels were not significantly different between the groups. However, expression of the half transporter *Abcg5* was increased in segment S2 and S3 of the intestine, however without any increase in the expression of the nuclear transcription factor LXRα in this segment. In contrast to the expression in liver, the expression of the cholesterol efflux transporter *Abca1* was increased approximately 3-fold in the two distal segments (Fig 5).

**Fig 6:**
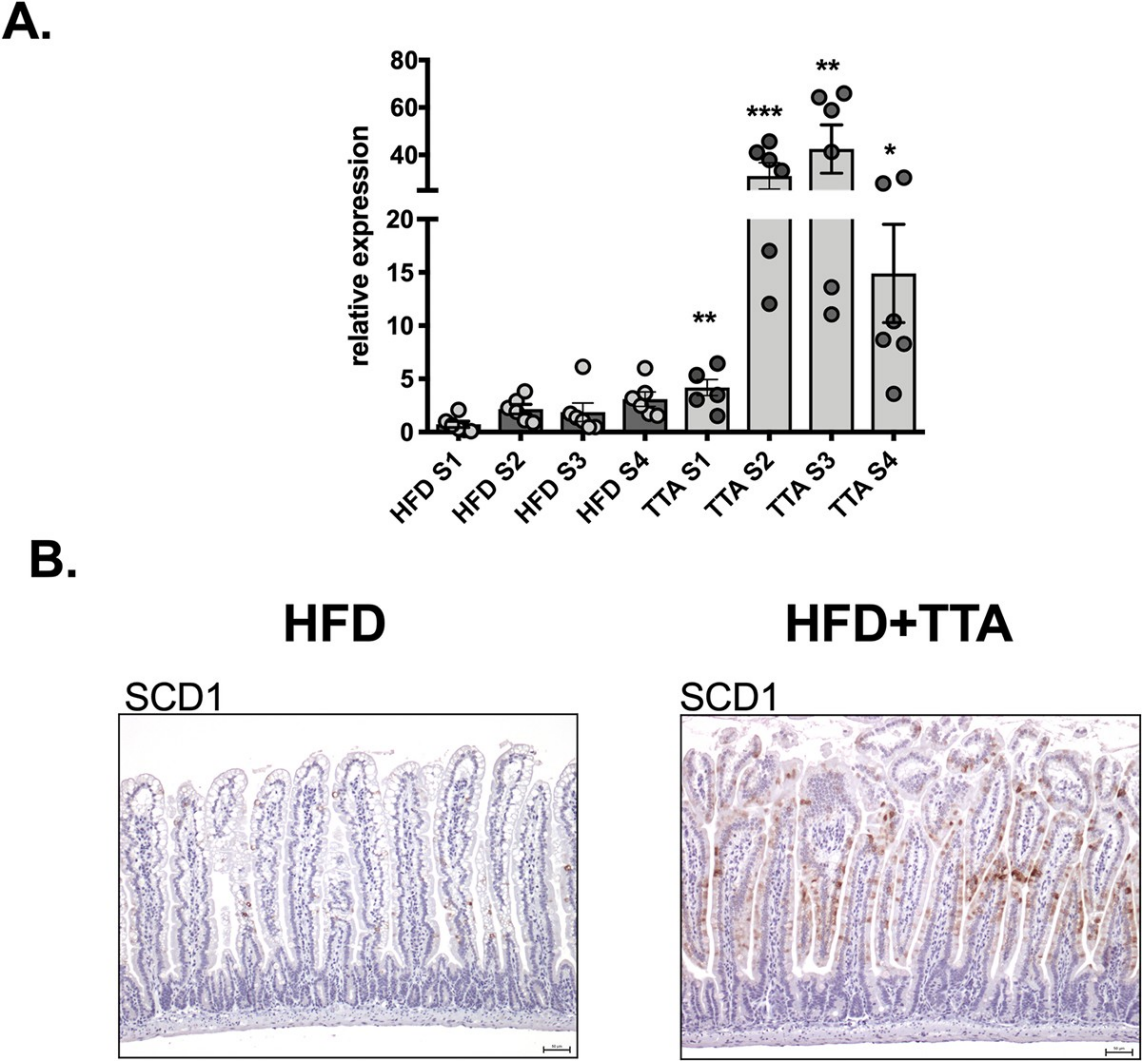
mRNA and protein expression of Scd1. **a.** mRNA expression of *Scd1* was analyzed on individual samples (n = 6) with S1 of one individual in the HFD control group used as a calibrator. Data in bar plots are shown as mean ± SEM, in HFD as dark grey bars with individual values in light grey circles and in HFD+TTA as light grey bars with individual values in dark grey circles and student´s unpaired t-test (two tailed) was performed, * = p<0.05 and ** = p<0.01. **b.** Immunohistochemistry of the middle part of the intestine using an antibody against SCD1. Left panel, HFD control and right panel, HFD+TTA.

Stearoyl-CoA desaturase 1(*Scd1*) mRNA expression was increased in all segments of the small intestine with the increase being most pronounced in the S2 and S3 segments (20–40 fold) in TTA treated animals (Fig 6A). Using an SCD1 specific antibody, immunoreactivity was mainly detected in enterocytes in the middle part of villi with the labeling being positive from jejunum (approximately S2) to the start of ileum (approximately S4) in intestines from the HFD control group. The labeling was weak and detected only in some cells. The increased expression of *Scd1* mRNA was translated into a robust increase in SCD1 protein in TTA fed mice with markedly increased immunoreactivity in the part of the intestine corresponding to the jejunum, with labeling further extending along the villus length although the strongest labeling was still evident in the middle part of villi (Fig 6B).

Strong positive SCD1 immunoreactivity was also detected in enterocytes overlying the lymphoid patches, but the expression was not affected by the TTA treatment (see S1 Fig).

## Discussion

To date only one study have investigated the beneficial effect of TTA in the gut: TTA administration reduced colonic wall thickening and the abundance of inflammatory cytokines in a dextran sodium sulfate induced model of colitis in rats [16]. However, the effect of TTA on the intestinal lipid metabolism and its possible contribution to modulation of plasma lipoprotein content have not been investigated. In the present study we aimed to investigate TTA effects on the intestinal and hepatic lipid and lipoprotein metabolism in mice fed HFD. TTA effectively reduced plasma TAG and attenuated body weight gain. This effect was coupled with a moderate increase in liver weight in accordance with previous findings of studies on TTA [8,12]. TTA’s effects on genes involved in hepatic mitochondrial and peroxisomal β-oxidation of fatty acids has earlier been show. The present study confirms and expand these findings to comprise genes across the entire pathways of mitochondrial and peroxisomal lipid metabolism. The regulation of these gene sets by TTA closely resembles those induced by other PPARα agonists with classical PPARα target genes being induced, such as *Ehhad* [27], and some genes being negatively regulated by PPARα activation, such as e.g. *Nudt7α* [28].

Despite the strong induction of genes involved in hepatic β-oxidation of fatty acids by TTA, it was evident that the levels of TAG increased in the liver, which is in contrast to the effects reported for most known PPARα agonists in the setting of fatty liver [29–34] However, increased liver TAG levels have earlier been found in rodents fed diets supplemented with PPARα agonists, [35–37],in overweight humans with NAFLD treated with fenofibrate [38] as well as in HFD fed hTNFα transgenic mice supplemented with TTA [39]. In this latter study the hepatic TAG accumulation were explained to be a dose and time dependent effect of the TTA supplementation [39,40]. TTA is a PPAR-pan agonist, thus also able to activate PPARγ. PPARγ ligands are known to cause mild steatosis accompanied by an increase in endogenous lipogenesis and expression of fatty acid transporters (for review, see [41]). Change in hepatic gene expression of classical lipogenic enzymes (i.e *Acaca* and *Fasn*) were however not found in these TTA-treated animals. Since no change in food intake was detected, while we could observe a reduction in white adipose tissue at the time of sacrifice (as also reported in [12]), the increased hepatic TAG levels in TTA-treated mice might be secondary to increased peripheral lipolysis and a consequent increase in hepatic uptake of plasma fatty acids. We could indeed show increased lipase activity in skeletal muscle, increased phosphorylation of HSL in white adipose tissue and increased expression of the fatty acid transporting and activating proteins FATP2 and ACLS1 in the liver of TTA-treated mice. Nevertheless, in our experimental setting, we could not rule out the possibility that a reduced hepatic VLDL secretion could have a concomitant causative effect. We indeed observed a reduction of both TAG and cholesterol in VLDL/remnants. This effect was coupled to a reduced amount of plasma ApoB (data not shown) in the TTA treated group. We did not observe any changes in hepatic *Mttp* and *ApoB* mRNA levels thus suggesting that TTA rather might affect the rate of lipidation and/or assembling of VLDL particle.

Further studies are required in order to elucidate these mechanisms that could cause liver TAG accumulation in mice secondary to TTA treatment.

Liver total cholesterol was unaffected by TTA treatment. This was in line with the unchanged mRNA levels of the enzymes *Hmgcr1* and *Cyp7a1* that are rate-limiting for cholesterol synthesis and its conversion into bile acids (Fig 3B). Notably, PPARα agonists are generally suppressors of *Cyp7a1* [42]. These results suggest that the effect of TTA on overall cholesterol metabolism in the liver is modest under the conditions used here.

We further investigated the effect of TTA treatment on plasma lipoprotein composition. TAG was reduced in both VLDL/remnants and LDL particles in agreement with previous studies in Wistar rats [6,43]. Mentioned studies revealed that TTA was also effective in reducing plasma cholesterol. However, in the present study performed in mice we clearly show that total plasma cholesterol levels are not changed by the TTA treatment, but that TTA instead causes changes in the composition of plasma lipoproteins resulting in the redistribution of cholesterol between the particles. TTA led to a reduction of plasma apoA-I, thus suggesting and atherogenic profile. On the other end, the levels of apoAI containing nascent pre-β HDL were increased by the treatment as most likely the results of the increased lipolysis observed in the extra hepatic tissues. Pre-β HDL seem to play, at least in vitro and in preclinical studies, an important role in anti-atherogenic the reverse cholesterol transport (RCT) pathway being an efficient acceptor of unesterified cholesterol from peripheral cells [44]. However, prebeta-1 HDL associates positively with the presence of CAD, [45–48], myocardial infarction [45], and carotid intima media thickness [49,50]. Moreover and in line with previous preclinical studies on fibrates [51], our results showed that TTA treatment led to the appearance of triglyceride poor and cholesterol rich large apoE containing HDL particles, having a density in the range of LDL, and a size in between the LDL and HDL particles. Interestingly, large apoE-HDL seem to be a diet-responsive metabolic pathway that renders HDL more biologically active in RCT [52].

These lipoprotein changes were coupled to a marked induction of the *Abca1* transporter mRNA in the small intestine, but not in the liver. ABCA1 is known for its importance in the formation of HDL in the small intestine [53]. Several studies have shown that PPARα agonists can induce expression of the *Abca1* gene in the small intestine, possibly through an indirect mechanism that may involve the LXR nuclear receptors [54–57]. Interestingly, other studies demonstrated increased plasma HDL cholesterol levels and increased levels of *Abca1* and *ApoA1* mRNA levels in the small intestine when a dual PPARα/δ agonist was employed in ApoE2-KI mice, which has a similar response to PPARα agonists as humans [58]. In the same study it was also shown that treatment of human jejunal explants with a PPARα/δ dual agonist induced both *Abca1* and *ApoA1* mRNAs. Since no effects on hepatic *Abca1* or *ApoA1* expression were observed in the present study it may be hypothesized that the apparent increase in ApoE-containing HDL fraction in TTA treated mice could possible improve cholesterol efflux from extrahepatic tissue and may as well play an anti-inflammatory role [59–62].

Despite the vast use of HFD in metabolic research relatively little is known about the effects of these diets on small intestine biology. Recently it has come to attention that enterocytes of the small intestine accumulate and store lipids when present in excess in the diet [63–65]. Lipid droplets were evident in enterocytes of HFD fed mice while the small intestines of the TTA treated animals displayed drastically reduced lipid content as seen from the histological analysis. This is in line with a previous study where it was shown that mice receiving HFD diet together with fenofibrate had reduced lipid content in the enterocytes compared to controls [66].

The actual absorption of lipids in the small intestine was not measured in the present study. However, if there was a reduction of actual lipid uptake under conditions used here, it occurred despite of an apparent increase of the absorption capacity of lipids in the small intestine of TTA treated mice, as indicated by markedly increased mRNA expression of *Cd36* and *L-Fabp*, genes involved in fatty acid transport were induced by TTA.

Mice fed HFD together with PPAR agonists fibrates or DHA display a reduced postprandial hypertriglyceridemia [57,66,67]. In conjunction to these findings it was demonstrated that mice fed HFD and fenofibrate have a substantially larger fecal loss of lipids [57,66].

Therefore, the induction of genes by TTA involved in lipid processing in the small intestine may in part be a response to a primary fecal loss of lipids. This should be addressed in detail in future studies of TTA.

Peroxisome proliferators have previously been shown to regulate expression of the *Scd1* gene in liver [68,69] and in the small intestine [54,70]. Upregulation of *Scd1* gene expression in the liver by TTA resulted in increased oleic acid content in liver and VLDL as well as accumulation of a delta 9-desaturated metabolite of TTA [1]. Interestingly, TTA induced expression of the *Scd1* gene at mRNA level to a much higher extent in small intestine than in the liver. The increased mRNA expression in the small intestine was translated into a markedly increased protein expression as shown by immunohistochemistry. The impact of elevated SCD1 expression in the small intestine under the challenge of HFD in the presence of TTA is at present not clear. However, it has been suggested that SCD1 is crucial for synthesis of cholesterol esters and triglycerides at least in liver [71]. Recently, it has been described that knockdown of Scd1 in on a *Ldlr*-/- background resulted in an altered plasma lipid pattern [72]. Therefore, increased expression of SCD1 in the small intestine may enhance the formation of chylomicrons and HDL with a subsequent release from the enterocytes by enhancing neutral lipid formation, which may contribute to the decreased lipid droplet size seen in enterocytes from the TTA treated mice. TTA has previously been shown to increase the amount of oleic acid in lipoproteins [1,30]. If an induction oleic acid production in the intestine is responsible for the altered plasma HDL pattern observed in this study remains to be investigated.

Additionally, it has been demonstrated that deletion of *Scd1* in the intestine increases intestinal inflammation and tumor burden in a colorectal and intestinal cancer mouse model [73]. This is interesting since SCD1 is known as major promoter of cancer cell survival in several, but not all cancer forms [74,75].

An additional interesting finding from the examination of SCD1 staining in the intestine was the positive staining in enterocytes overlying the lymphoid patches in the intestine, however, the staining of these cells was apparently not affected by the TTA treatment.

In summary, this work supports the notion of TTA as an efficient activator of intestinal and hepatic expression of genes for fatty acid uptake and oxidation. There is also a tissue specificity in the regulation of certain genes by TTA, i.e. *Acot1* displayed higher increase in the liver than the small intestine while the opposite was found for *Scd1*. TTA also induced a shift in the plasma cholesterol profile with larger HDL particles and less LDL. The TTA mediated effects on lipid metabolism in the small intestine such as a dramatically reduced enterocyte lipid content are likely to be of importance for the overall systemic effects of TTA on lipid homeostasis. The finding of TTA as a lipid lowering drug in humans [7] prompts for more detailed studies of the biological actions of TTA and TTA derived molecules in the processes of lipid metabolism in the small intestine.

## Supporting information

S1 Fig SCD1 in lymphoid patches. Immunohistochemistry of the small intestine showing immunolabeling of SCD1 in cells surrounding lymphoid patches of a HFD control mouse.
(TIFF)

## Abbreviations

CM: chylomicrons
HDL: high density lipoprotein
HFD: high fat diet
LDL: low density lipoproteins
PL: phospholipid
PPAR: peroxisome proliferator activated receptor
SCD1: steoryl-CoA desaturase 1
SEC: size exclusion chromatography
TAG: triacylglycerol
TTA: tetradecylthioacetic acid
VLDL: very low density lipoproteins
