# Supplementary figures and images for: The PPAR pan-agonist tetradecylthioacetic acid promotes redistribution of plasma cholesterol towards large HDL
Source: PLoS One. 2020 Mar 16;15(3):e0229322. doi: 10.1371/journal.pone.0229322 (PMC7075573; doi:10.1371/journal.pone.0229322)

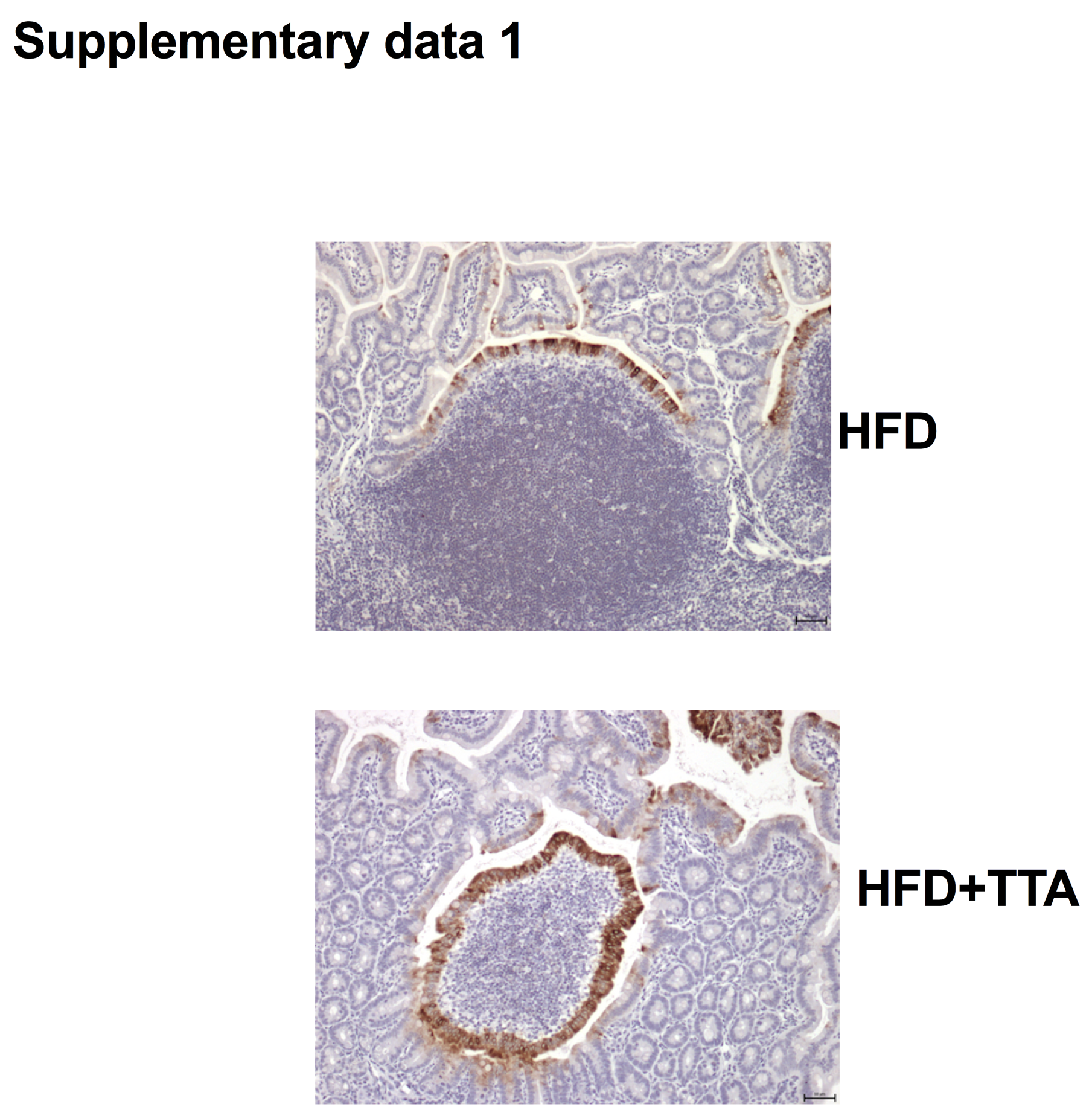

Supplement: S1 Fig — Immunohistochemistry of the small intestine showing immunolabeling of SCD1 in cells surrounding lymphoid patches of a HFD control mouse. (TIFF) [file pone.0229322.s001.tiff]
